# The Safety and Impact of a Model of Intermittent, Time-Restricted Circadian Fasting (“Ramadan Fasting”) on Hidradenitis Suppurativa: Insights from a Multicenter, Observational, Cross-Over, Pilot, Exploratory Study

**DOI:** 10.3390/nu11081781

**Published:** 2019-08-01

**Authors:** Giovanni Damiani, Naim Mahroum, Paolo Daniele Maria Pigatto, Alessia Pacifico, Piergiorgio Malagoli, Danica Tiodorovic, Rosalynn RZ Conic, Howard Amital, Nicola Luigi Bragazzi, Abdulla Watad, Mohammad Adawi

**Affiliations:** 1 Department of Dermatology, Case Western Reserve University, Cleveland, OH 44106, USA; 2 Young Dermatologists Italian Network, Centro Studi GISED, 24122 Bergamo, Italy; 3 Clinical Dermatology, IRCCS Istituto Ortopedico Galeazzi, 20161 Milan, Italy; 4 Department of Biomedical, Surgical and Dental Sciences, University of Milan, 20161 Milan, Italy; 5 Sackler Faculty of Medicine, Tel-Aviv University, Tel-Aviv 6997801, Israel; 6 Clinical Dermatology Department, San Gallicano Dermatological Institute, IRCCS, 00144 Rome, Italy; 7 Dermatology Unit, Azienda Ospedaliera San Donato Milanese, 20097 Milan, Italy; 8 Department of Dermatology, University of Nis, 18000 Nis, Serbia; 9 Postgraduate School of Public Health, Department of Health Sciences, University of Genoa, 16132 Genoa, Italy; 10 Department of Mathematics and Statistics, Laboratory for Industrial and Applied Mathematics, York University, Toronto, ON M3J 1P3, Canada; 11 Department of Medicine B and Zabludowicz Center for Autoimmune Diseases, Sheba Medical Center, Tel-Hashomer, Ramat-Gan 5265601, Israel; 12 Section of Musculoskeletal Disease, Leeds Institute of Molecular Medicine, University of Leeds, NIHR Leeds Musculoskeletal Biomedical Research Unit, Chapel Allerton Hospital, Leeds LS7 4SA, UK; 13 Padeh and Ziv Hospitals, Azrieli Faculty of Medicine, Bar-Ilan University, Ramat Gan 5290002, Israel

**Keywords:** hidradenitis suppurativa, dietary intervention, intermittent fasting, circadian rhythms, human biological clock

## Abstract

Hidradenitis suppurativa (HS) is a chronic-relapsing and debilitating disease, which affects the components of the folliculopilosebaceous unit and severely impacts on the perceived health-related quality of life. Among the possible treatments, dietary interventions, such as fasting, have been described to positively impact on HS. However, nothing is known about the effects of circadian, intermittent fasting, such as the Ramadan fasting. A sample of 55 HS patients (24 males (43.6%) and 31 females (56.4%), mean age 39.65 ± 8.39 years, average disease duration 14.31 ± 7.03 years) was recruited in the present study. The “Severity of International Hidradenitis Suppurativa Severity Score System” (IHS4) decreased significantly from 11.00 ± 5.88 (before Ramadan) to 10.15 ± 6.45 (after Ramadan), with a mean difference of −0.85 ± 0.83 (*p* < 0.0001). At the univariate analyses, the improvement was associated with HS phenotype (with a prominent improvement among those with ectopic type), treatment (with the improvement being higher in patients receiving topical and systemic antibiotics compared to those treated with biologics), the “Autoinflammatory Disease Damage Index” (ADDI), and Hurley scores. At the multivariate regression analysis, only the Hurley score (regression coefficient = 0.70, *p* = 0.0003) was found to be an independent predictor of change in the IHS4 score after fasting. The improvement in the IHS4 score was not, however, associated with weight loss. In conclusion, the Ramadan fasting proved to be safe and effective in HS patients. Considering the small sample size and the exploratory nature of the present investigation, further studies in the field are warranted, especially longitudinal, prospective and randomized ones.

## 1. Introduction

Hidradenitis suppurativa (HS), also known as *acne inversa* or Verneuil’s disease, is a chronic, debilitating condition, affecting the components of the folliculopilosebaceous unit and surrounding tissue, such as hair follicles and apocrine sweat glands [1,2]. From a pathogenetic standpoint, the main processes which lead to HS are hyperkeratosis, inflammation, and perifolliculitis [3]. HS is clinically characterized by recurrent, relapsing episodes of swollen, deep-seated, and rather painful abscesses and nodules, hypertrophic scars, draining fistulae and sinus tracts. These generally involve the skin folds and other body areas, like axillary, inguinal, anogenital, and infra-mammary areas, where contacts, pressures, shearing forces, and frictions are frequent [2,3,4]. 

The pathogenesis of HS is complex, multi-factorial and still poorly understood, with the biological make-up of the individual (especially, genetic polymorphisms, endocrinological drivers and hormones), as well as epigenetic factors and environmental interactions playing a major role [4]. HS is a systemic, autoinflammatory condition [5] since several disorders, including autoimmune/autoinflammatory conditions, have been shown to be associated with HS, like inflammatory bowel disease (IBD) and spondyloarthropathies [6], non-alcoholic fatty liver disease (NAFLD) [7] or neutrophilic interstitial pneumonia [8], among others. 

HS affects more commonly women, with a female to male ratio of 2–3/1 and with a prevalence variable from 0.1% to 4%, depending on the investigated population and the adopted methods and study design [9].

Concerning the treatment [10], different pharmacological options are available, ranging from antibiotics (such as clindamycin, rifampicin or tetracyclines) to biologics (like Adalimumab or Infliximab) [11,12,13]. However, drugs have side-effects and are, in some cases, interrupted, being not well-tolerated by patients [10]. 

Considering non-pharmacological treatment [14], surgery represents another possible way of managing HS. Dietary interventions—such as restraining from consuming dairy products or foods with high glycemic index–have been reported as beneficial, and can positively impact on HS severity [14,15,16,17]. A proper diet seems to counteract the biological processes of over-expression of cytokeratins, hyperproliferation of keratinocytes, hypercornification of the follicular wall, and inflammation, which, as previously mentioned, are typical of HS [17]. However, these reports are often scarce or anecdotal, with a dearth of information concerning, for example, the potential impact of intermittent circadian fasting. 

The Ramadan fasting is a rather popular model of intermittent circadian fasting that is practiced every year by the Muslim population worldwide [18]. A recent review [19] has shown that this kind of fasting has positive immunomodulatory effects on skin diseases, as well as on rheumatic disorders [20,21,22].

Given the paucity of data specifically concerning HS, the present pilot exploratory study was aimed to fill this gap in knowledge, in order to shed light on the potential contribution of dietary manipulation in modulating HS-related systemic inflammation. 

## 2. Materials and Methods

### 2.1. Ethical Clearance and Study Design

This study represents a sub-analysis of a previous observational study approved by the Milano Area 2 Ethical Committee on 18 December 2017 with the approval unique number ID 804_2017. 

The study protocol was reviewed in-depth and received full approval by the Ethical Committee of each center which participated in the study (namely, the Dermatology Unit of the hospital IRCCS Istituto Ortopedico Galeazzi, Milano, Italy; the hospital IRCCS San Donato, Milan, Italy; the IRCCS San Gallicano Dermatological Institute, Italy; and the Department of Dermatology, University of Nis, Nis, Serbia).

The present study was conducted in accordance with the 1964 Helsinki ethical declaration and its subsequent amendments. 

It is important to stress that the Islamic medicine exonerates sick people from the observance of the Ramadan fasting and that all subjects which took part in the present investigation volunteered to observe the fast, under strict medical monitoring and checks. Nobody was forced to fast and, as such, this study is not an interventional, non-pharmacological, non-randomized trial, but rather a multi-center, observational, cross-over study. It is of crucial importance to notice that: (i) it is extremely difficult to recruit adequate controls for cases observing the fast, in that they should be matched for age, gender, disease severity and ethnicity; and (ii) cross-over studies (in which cases act as controls of themselves) are not immune from the carry-over effect.

For this reason, the statistical analysis focused mainly on two time-points (before and at the end of the Ramadan fasting). Data regarding 1 month after the Ramadan are reported as exploratory and merely indicative of the temporal trend of the variables under study. Further time-points should be considered in future longitudinal, prospective studies.

However, on the other hand, no specific indications exist about the impact and safety of Ramadan on HS, therefore this study was designed as pilot, exploratory investigation, aimed at following-up HS patients under stable pharmacological treatments that refused to skip the Ramadan fasting as recommended by their dermatologists.

The present study took place in the period 16 May 2018–14 June 2018 and data were collected retrospectively.

### 2.2. Patient Selection: Inclusion and Exclusion Criteria

Before the enrollment, patients underwent a preliminary self-screening with visual-aided questionnaire for the self-assessment of HS, which was further confirmed by dermatologists [23].

Patients were included if: (i) aged 18 years or greater, (ii) accepted to sign a written, informed consent, (iii) being diagnosed with HS by two independent experienced (≥10 years of clinical practice), board-certified dermatologists, (iv) willing to completely observe the Ramadan fasting every day, from dawn to sunset, during the year 2018; (v) with no drug abuse and with an “Alcohol Consumption Screening Questionnaire” (AUDIT) score less than 7; (vi) receiving biologics in maintenance and not in induction phase; and (vii) receiving other HS-related drugs since at least 4 weeks before the commencement of the Ramadan fasting. 

Patients were excluded if they were: (i) aged less than 18 years, (ii) undergoing HS surgery, (iii) pregnant or breastfeeding women, (iv) suffering from primary or secondary immunodeficiencies, such as HIV/AIDS, (v) being seropositive for hepatitis C virus (HCV) or hepatitis B virus (HBV), (vi) suffering from tuberculosis or other chronic infections; (vii) suffering from inflammatory/autoimmune co-morbidities (including IBD, psoriasis, psoriatic arthritis or systemic lupus erythematosus, SLE), (viii) suffering from any medical issue preventing the observance of the fasting, and (ix) willing to only partially fast or to completely refrain from observing the Ramadan fasting.

### 2.3. Socio-Demographic and Clinical Measures

For each patient, the following socio-demographic and clinical parameter were recorded: age, gender, country of origin, smoking status, drug history, family history of HS and of autoimmune diseases, HS phenotype [24] and eventual co-morbidities. A subject was considered ex-smoker if he/she had quit smoking at least 10 years before the study took place.

Furthermore, the treatment received was recorded. It should be noted that oral or injectable drugs were taken during the night in order not to break the fasting, while topical drugs were also consumed during the day.

Disease severity of HS was quantitatively assessed computing both static and dynamic indexes. The former indicators comprised the “Autoinflammatory Disease Damage Index” (ADDI) score [25] and the Hurley score [26], while the latter included the “Severity of International Hidradenitis Suppurativa Severity Score System” (IHS4) [27]. The IHS4 was recorded as well as the exact number of the counted lesions, namely inflammatory nodules, abscesses and draining fistulas, in order to increase the sensibility of the assessment, since inflammatory nodules are recurrent and transitory as well as abscesses, whereas draining fistulas have a longer life and are expression of a higher level of chronic inflammation. The IHS4 score was calculated 1–3 days before the beginning of the Ramadan fasting, and 1–3 days after the end of the month of Ramadan. Based on the IHS4 score, patients were classified into mild (≤3 points), moderate (4–10 points) and severe (≥11 points) HS patients [27]. 

### 2.4. Statistical Analysis

Data were visually inspected before commencing any data processing, in order to capture potential outliers. Furthermore, normality of data distribution was verified carrying out the Shapiro-Wilk’s test, which was preferred to other tests (such as the Kolmogorov-Smirnov or the D’Agostino-Pearson *omnibus* tests) given the small sample size utilized in the present investigation. Continuous variables were normally distributed and, as such, were computed as means ± standard deviation, whereas categorical parameters were expressed as percentages, where appropriate.

Change in the IHS4 score before and after Ramadan was assessed carrying out the Student’s paired *t*-test, while associations between change in the IHS4 score and other variables under study (like age, disease duration, the Hurley and ADDI scores) were evaluated by means of the Pearson’s correlation. The magnitude of the correlation coefficient was interpreted according to the following rule of thumb: the correlation was deemed to be negligible if the r coefficient ranged from 0.00 to 0.30, was deemed to be low with r in the range 0.30–0.50, was judged moderate with r from 0.50 to 0.70, high with r in the range 0.70–0.90, and, finally, very high with r in the range 0.90–1.00.

The impact of different variables (gender, HS phenotype, treatment received–topical and systemic antibiotics, or biologics) on change in the IHS4 score was investigated conducting the Student’s *t*-test (between two groups) or the analysis of variance (ANOVA, for more than two groups). The overall effect of the Ramadan fasting was evaluated with repeated measures ANOVA (rmANOVA)).

It should be emphasized that from a pharmacological standpoint, not all antibiotics used in the management of HS have the same mechanisms of action and effectiveness. However, given the small sample size, once verified that no significant differences could be found for the various antibiotics, we preferred to pool data together, focus on the route of administration (topical *versus* systemic) and considering apart biologics. The precise impact of the different types of antibiotics in the treatment and management of HS and its interaction with the dietary intervention warrants further studies. 

Furthermore, a multivariate regression analysis was conducted to shed light on the determinants of change in the IHS4 score. Since the number of variables under study was higher than the number expected *a priori* based on the sample size and applying the rule of thumb of 1 predictor per 10 subjects, the best variables were chosen based on different regression models being run and selected according to their goodness-of-fit and quality indicators. 

Considering nodules, abscesses and draining tunnels, given their nature of count variables, multivariate log-linear robust Poisson models were carried out. 

Statistical analyses were carried out with the commercial software “Statistical Package for Social Sciences” (SPSS version 24.0 for Windows, IBM, Armonk, NY, USA). Graphs were generated utilizing the commercial software MedCalc (version 18.11.3, MedCalc Software bvba, Ostend, Belgium). 

For all statistical analyses, figures with a *p*-value < 0.05 were considered statistically significant, except for those cases in which the Bonferroni’s correction was applied as *post-hoc* test in order to ensure protection against multiple testing. 

## 3. Results

### 3.1. Descriptive Statistics

**Figure 1:**
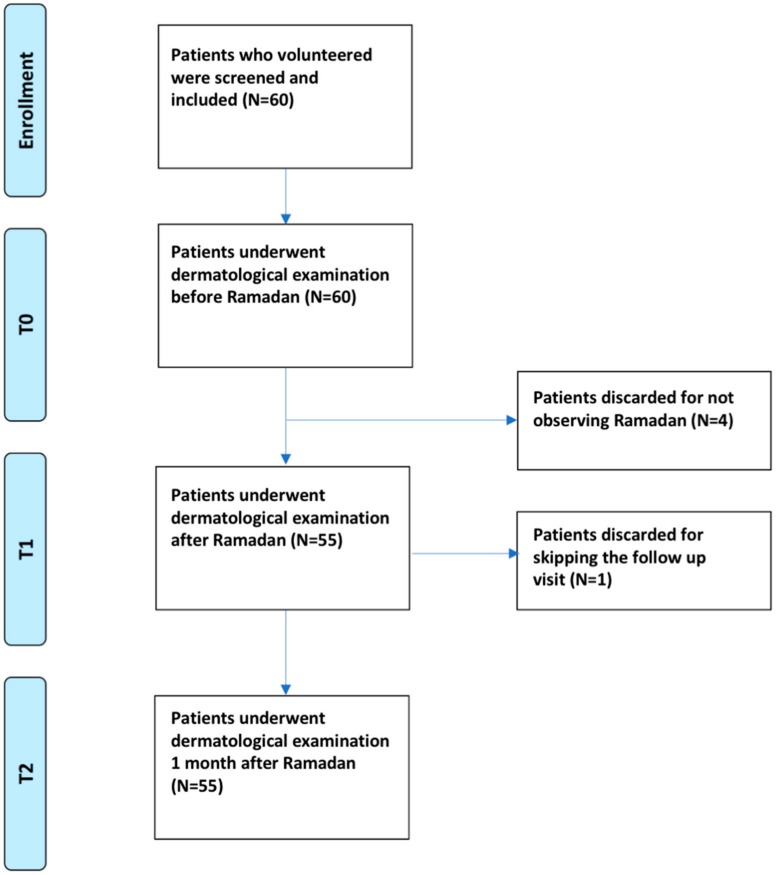
The study flow diagram, showing the different steps and time-points of the investigation (T_0_ before the beginning of the month of Ramadan, T_1_ after the end of the Ramadan fasting, and T_2_ one month after the fasting month).

A sample of 60 subjects volunteered to participate, 5 individuals were excluded, based on the above-mentioned inclusion and exclusion criteria (*n* = 4, because they did not completely observe the Ramadan fasting, *n* = 1, because the patient did not attend the last visit and was lost at the follow-up) (Figure 1).

Finally, 55 patients were recruited in the present study. Twenty-four (43.6%) were males, whereas 31 (56.4%) were females. Mean age was 39.65 ± 8.39 years (range 21–56 years). Seventeen (30.9%) patients had a family history of HS and 21 (38.2%) a family history of autoimmune disorders (Hashimoto’s thyroiditis, *n* = 7, SLE, *n* = 2, rheumatoid arthritis, *n* = 1, Crohn’s disease, *n* = 5, ulcerative colitis, *n* = 2, Sjögren’s syndrome, *n* = 2, and undifferentiated connective tissue disease, *n* = 2). 

Most patients were from Turkey (*n* = 16, 29.1%), whereas 9 (16.4%), 8 (14.5%), and 7 (12.7%) subjects were from Pakistan, Morocco, and Senegal, respectively. A further 3 (5.5%) and 2 (3.6%) were from Kazakhstan and Jordan, respectively. Other 2 (3.6%) individuals were from Sudan, whereas the reminders were from Bosnia and Herzegovina (*n* = 1, 1.8%), Cameroon (*n* = 2, 3.6%), Cyprus (*n* = 1, 1.8%), France (*n* = 1, 1.8%), Germany (*n* = 1, 1.8%), Greece (*n* = 1, 1.8%), and India (*n* = 1, 1.8%). Thirty-four (61.8%) were first-generation migrants, whereas 20 (36.4%) and 1 (1.8%) were of second and third generation, respectively. 

Twenty-four (43.6%) subjects were current smokers, whereas 8 (14.5%) were ex-smokers and 3 (5.5%) smoked only e-cigarettes. Concerning co-morbidities, 8 (14.5%), 5 (9.1%), 4 (7.3%) and 1 (1.8%) patients suffered from dyslipidemia, osteoporosis, steroid resistant asthma, and cerebro-vascular accident. 

Average disease duration was 14.31 ± 7.03 years. Concerning HS phenotype, 24 (43.6%), 8 (14.5%), 8 (14.5%), 6 (10.9%), 6 (10.9%) and 3 (5.5%) suffered from regular, scarring folliculitis type, frictional furuncle type, conglobata type, ectopic and syndromic HS phenotypes, respectively. 

From a clinical standpoint, before the month of Ramadan 24 patients had at least 1 draining fistula and 51 had at least 1 abscess.

Regarding the static indexes of disease severity, the Hurley score was 2.31 ± 0.77, whereas the ADDI score resulted 1.69 ± 0.96. More in detail, 10 (18.2%), 18 (32.7%) and 27 (49.1%) patients had a Hurley score of 1, 2 and 3, respectively. Before the month of Ramadan, based on the IHS4 score, 6 (10.9%), 22 (40.0%) and 27 (49.1%) patients suffered from mild, moderate and severe HS, respectively. 

Concerning changes in weight before and after the Ramadan fasting, overall median value was 0 kg, meaning that no changes in weight could be recorded. More specifically, 12 (21.8%) individuals and 1 (1.8%) subject lost 1 and 2 kg, respectively, whereas 9 (16.4%) and 1 (1.8%) patients acquired 1 and 2 kg, respectively. Thirty-two (58.2%) individuals did not lose any kg.

Regarding the pharmacological treatment, twenty-three (41.8%) patients were on biologics (Adalimumab (40 mg each week in maintenance phase), *n* = 22; Ustekinumab (90 mg in maintenance phase from week 16 each 12 weeks), *n* = 1), whereas 24 (43.6%) subjects were treated with systemic antibiotics (clindamycin (300 mg b.i.d. alone or in combination with rifampicin 600 mg once daily up to 10 weeks), *n* = 2, rifampicin (600 mg once daily for 10 weeks), *n* = 11, and tetracyclines (500 mg b.i.d. for up to 4 months), *n* = 9). 

The remaining 8 (14.5%) patients received topical antibiotics (clindamycin (twice daily for 3 months)). 

### 3.2. Changes in the IHS4 Score: Univariate Analyses

The overall effect of fasting on the IHS4 score was statistically significant (F = 41.61, *p* < 0.0001). Remarkably at the end of Ramadan, 35/51 (68.6%) patients experienced a decrease in the number of abscesses and 9/24 (37.5%) experienced a decrease in the number of draining fistulas, whilst 1 month after the end of Ramadan, only 14/55 (25.5%) and 5/24 (20.8%) maintained the beneficial effect of the fasting. No medication changes were performed during the study period.

**Figure 2:**
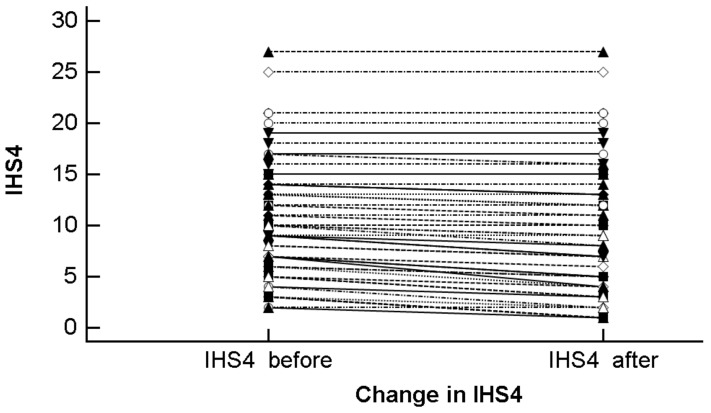
Change in the IHS4 score before (T_0_) and after the Ramadan fasting (T_1_).

Considering the time-points T_0_ and T_1_, the IHS4 score decreased significantly from 11.00 ± 5.88 to 10.15 ± 6.45 (mean difference −0.85 ± 0.83 (95% CI −1.08 to −0.63), *t* = −7.67, *p* < 0.0001), as shown in Figure 2. At the end of the month of Ramadan (at T_1_), based on the IHS4 score, 10 (18.2%), 19 (34.5%) and 26 (47.3%) patients suffered from mild, moderate, and severe HS, respectively.

No differences could be found according to gender (delta = −0.94 ± 0.89 for females, delta = −0.75 ± 0.74 for males). Similarly, no differences could be detected concerning family history for HS (delta = −0.97 ± 0.79 in absence of familiarity and delta = −0.59 ± 0.87 in presence of familiarity). 

**Figure 3:**
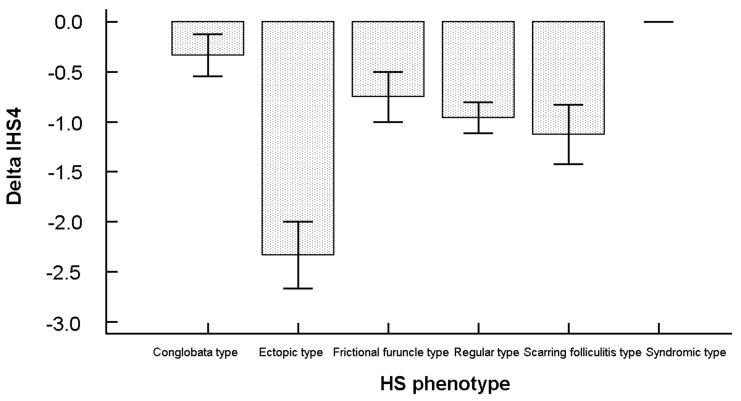
Change in the IHS4 score before (T_0_) and after (T_1_) Ramadan broken down according to the HS phenotype.

A significant correlation was found between delta in the IHS4 and ADDI scores (r = 0.34 (95% CI 0.08–0.55), *p* = 0.0116, low correlation), as well as between delta in the IHS4 and Hurley scores (r = 0.66 (95% CI 0.48–0.79), *p* < 0.0001, moderate correlation). Correlations with age or with disease duration failed, instead, to reach statistical significance. Similarly, the correlation between delta in the IHS4 score and delta in weight was not statistically significant. Stratifying according to the HS phenotype, there was an overall significant difference among the types (F = 5.67, *p* < 0.0001). More specifically, the type which changed the most before and after Ramadan was the ectopic one (delta = −2.33 ± 0.58), whereas the syndromic one did not change, as shown in Figure 3. 

**Figure 4:**
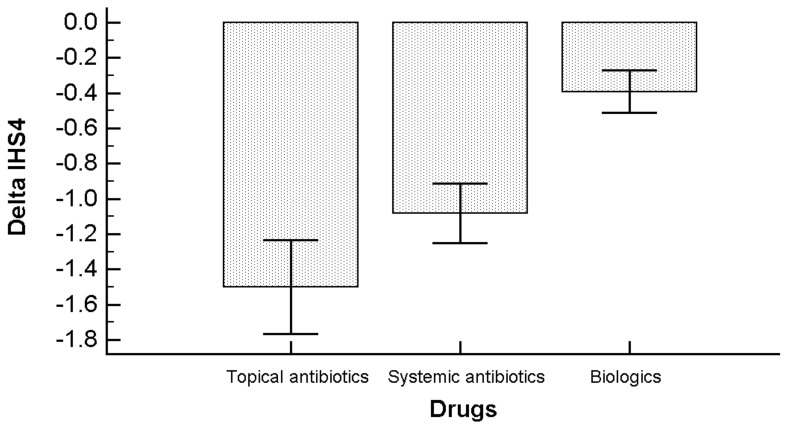
Change in the IHS4 score before (T_0_) and after (T_1_) Ramadan broken down according to the treatment received.

Concerning the pharmacological treatment, the greatest changes in the IHS4 score could be observed for patients under topical and systemic antibiotics, whilst the least change was found for individuals receiving biologics (Figure 4). No side-effects could be reported.

### 3.3. Changes in the IHS4 score: Multivariate Analyses

At the multivariate regression analysis, as reported in Table 1, only the Hurley score (regression coefficient = 0.66, *p* < 0.0001) was found to be an independent predictor of change in the IHS4 score.

**Figure 5:**
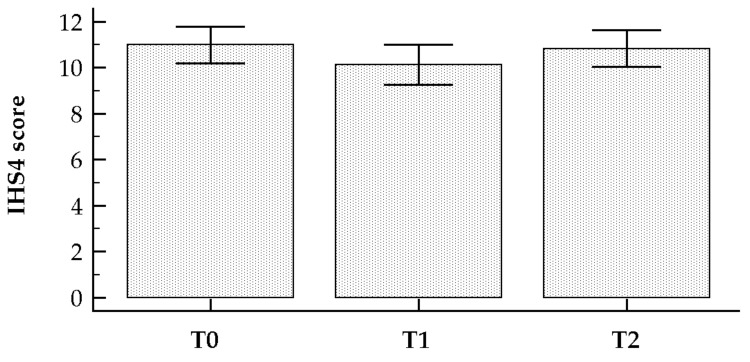
Change in the IHS4 score at the different time-points (T_0_ before the beginning of the month of Ramadan, T_1_ after the end of the Ramadan fasting, and T_2_ one month after the fasting month).

Considering also the third time-point (T_2_), the IHS4 score tended to return to its baseline value (10.84 ± 5.88), exhibiting, as such, a quadratic trend (t = 6.93, *p* < 0.0001) (Figure 5). 

### 3.4. Changes in Nodules: Multivariate Analyses

At the multivariate log-linear robust Poisson regression analysis, as reported in Table 2, HS phenotype (F = 5.33, *p* = 0.001), family history of HS (F = 9.31, *p* = 0.004) and the Hurley score (F = 8.07, *p* = 0.007) resulted predictors of changes in nodules, whereas disease duration (F = 2.01, *p* = 0.163) and change in weight (F = 1.78, *p* = 0.189) were borderline significant. More in detail, subjects with no family history of HS had a likelihood of 1.85 ((95% CI 1.35–2.54), *p* = 0.000) of reporting changes in nodules. Similarly, the Hurley score resulted an independent predictor (likelihood 1.55 ((95% CI 1.29–1.86), *p* = 0.000). Conversely, with respect to the syndromic type, all other HS phenotypes significantly did not report any changes in nodules (conglobata type 0.22 (95% CI 0.12–0.40), *p* = 0.000, ectopic type (0.36 (95% CI 0.21–0.61), *p* = 0.000, frictional foruncle type 0.27 (95% CI 0.16–0.45)), *p* = 0.000, regular type 0.46 (0.29–0.72), *p* = 0.001, and scarring folliculitis type 0.33 (95% CI 0.20–0.54), *p* = 0.000).

### 3.5. Changes in Abscesses: Multivariate Analyses

At the multivariate log-linear robust Poisson regression analysis, HS phenotype (F = 100.10, *p* = 0.000) and the Hurley score (F = 25.67, *p* = 0.000) resulted independent predictors of changes in abscesses, whereas a family history of HS (F = 1.18, *p* = 0.278), drugs (F = 3.14, *p* = 0.209) and a change in weight (F = 0.47, *p* = 0.493) failed to achieve a significance threshold. More in detail, with respect to syndromic type, subjects with frictional foruncle type and a scarring folliculitis type had a likelihood of 2.59 ((95% CI 1.75–3.83), *p* = 0.000) and 2.03 ((95% CI 1.43–2.87), *p* = 0.000) of reporting changes in abscesses, respectively. Similarly, the Hurley score (likelihood of 1.80 (95% CI 1.44–2.27), *p* = 0.000) was an independent predictor associated with changes in abscesses before, during and after the month of Ramadan. Further details are reported in Table 3. 

### 3.6. Changes in Draining Tunnels: Multivariate Analyses

At the multivariate log-linear robust Poisson regression analysis, HS phenotype (F = 118.58, *p* = 0.000) and the Hurley score (F = 6.35, *p* = 0.012) were associated with changes in draining tunnels. More in detail, the Hurley score resulted an independent predictor (likelihood of 1.26 (95% CI 1.05–1.51), *p* = 0.012). Conversely, with respect to syndromic type, subjects with an ectopic type (0.48 (0.33–0.72), *p* = 0.000), frictional foruncle type (0.30 (0.22–0.42), *p* = 0.000), regular type (0.46 (95% CI 0.33–0.65), *p* = 0.000), and scarring folliculitis type (0.28 (95% CI 0.20–0.38), *p* = 0.000) significantly did not report any changes in abscesses. More details are shown in Table 4.

## 4. Discussion

The present multi-center, observational, cross-over, pilot, exploratory study demonstrated that in HS patients, observing an intermittent fasting during the month of Ramadan has led to a significant improvement in the IHS4 score. This improvement was found to be associated with the HS phenotype, treatment received, and the ADDI and Hurley scores, but not with a change in weight. Despite the statistically significant difference in the IHS4 changes before and after the Ramadan fasting, its clinical improvement was modest but enough to maintain that the Ramadan fasting is not detrimental for HS patients.

Interestingly, despite the IHS4 score as absolute number not changing in a clinically significant manner, the amount of severe lesions (draining fistulas and abscesses) at the end of Ramadan decreased in 39/55 (70.9%) individuals and this finding may have a clinical value and implications. The nodules in our model changed depending on HS family history, disease duration, and HS phenotypes, suggesting that this dietary strategy may be more beneficial in particular subsets of patients. Remarkably, in 35/51 (68.6%) patients and 9/24 (37.5%), respectively, the number of abscesses and draining fistulas decreased after Ramadan but this benefit was maintained in only half of the subjects after 1 month. This data may support the idea that intermittent circadian fasting may have a different degree of beneficial effect depending on the disease severity.

HS has been shown to be a co-morbidity of obesity, diabetes and metabolic syndrome, with the severity of disease being linked to metabolic impairments [28,29]. From a molecular standpoint, a complex metabolic loop, involving the conserved serine/threonine kinase mammalian target of rapamycin (mTOR) and insulin resistance, could explain the insurgence of HS [30].

A number of epidemiological surveys seem to confirm this hypothesis. A recent cross-sectional, case-control study recruiting a sample of 137 subjects (76 HS patients *versus* 61 age- and gender-matched controls) found higher values of insulin resistance in cases. with respect to controls This association remained statistically significant, after adjusting for age, sex and body mass index (BMI), even though a correlation with disease severity could not be found [31]. Another case-control study [32] recruited 40 patients (with a male to female ratio of 23/17) and 40 age- and gender-matched controls. A significant association was found between HS and visfatin, insulin and C-reactive protein (CRP) levels, with each unit increase in visfatin and insulin increasing the risk of HS by 1.56- and 1.09-times, respectively. Finally, a recent meta-analysis of case-control studies addressing the relationship between HS and metabolic syndrome computed a crude odds-ratio (OR) of 1.95 (95% CI 1.31–2.89) and, after correcting for age, sex, other cardiovascular risk factors, and co-morbidities, an adjusted OR of 2.19 (95% CI 1.70–2.81) was obtained [33].

In our sample, we could not observe any change in weight. This finding is consistent with other studies about Ramadan fasting and with a recently published meta-analysis [34], and suggests that the improvement in the IHS4 score cannot be attributed to the loss of weight. 

Some transcriptomic studies have shown the role of circadian clock genes regulating hair follicles cycle [35] and the activity of apocrine sweat glands [36] in the etiopathogenesis of HS [37]. We could speculate that the improvement in IHS4 score could be due to the activation of a pool of circadian genes, since the Ramadan fasting alternates periods of feeding with moments of fasting according to the lunar calendar [18]. 

However, the extent of the contribution of circadian factors to the development of HS has been rarely explored and warrants further investigations. 

Another mechanism could be the immunomodulatory effect of the Ramadan fasting [18]. HS is a T helper 1 (Th1)-, T helper 17 (Th17)-mediated disorder, characterized by the up-regulation of various cytokines such as tumour necrosis factor-α (TNF-α) and Th17/interleukin-23 (IL-23) [38,39,40,41]. A murine model study has shown that diet mimicking fasting can increase the number of regulatory T (T_reg_) cells, while significantly reducing the levels of pro-inflammatory cytokines, decreasing the numbers of Th1, Th17 cells, and antigen-presenting cells (APCs), thus counteracting the effects of auto-immunity and auto-inflammation [42]. 

Remarkably, patients under biologics had the smallest improvements because they originally had a more severe disease. With regard to abscesses and draining fistulas that are consolidated, and deep seated lesions due to chronic inflammation, dietary interventions may have a lower impact than in nodules that are expression of acute inflammation and incipient HS flare(s). Since draining fistulas are lesions that deeply change the tissue architecture, the expected contribution of dietary regimen is minimal, lower than the impact observed, instead, on abscesses that less affect tissue architecture and sensibly lower than the effect on nodules that only minimally compromise the tissue architecture. 

Noteworthy, syndromic patients had higher level of systemic inflammation and may benefit less from fasting, whereas patients with frictional furuncle and scarring folliculitis types that had a lower level of systemic inflammation consequently may benefit more from dietary interventions. 

### Strengths and Limitations

The present study has some strengths, including its novelty and the thorough characterization of HS patients, taking into account also their phenotypes, which are generally overlooked by researchers [43]. To the best of our knowledge, it is the first investigation assessing the impact of dietary interventions on HS phenotypes, besides the generic weight loss. 

On the other hand, this study suffers from a number of limitations which should be properly acknowledged. During the month of Ramadan people do not observe usual working rhythms so in order to minimize this environmental confounder, we decided to follow-up the subjects twice after Ramadan, at the end of the fasting month (T_1_) and after 1 month (T_2_). The major shortcoming is represented by the small sample size and by the lack of controls. Therefore, concerning the study design, high-quality longitudinal, prospective, randomized studies are urgently needed. Recently two study protocols have been published, with the purpose of systematically studying the effect of dietary components in patients with chronic autoinflammatory diseases, including HS [44,45].

## 5. Conclusions

This study showed that the Ramadan fasting proved to be safe and effective in HS patients. However, given the above-mentioned shortcomings, further research in the field is warranted, especially longitudinal, prospective, and randomized studies.

## Figures and Tables

**Table 1:** Multivariate regression analysis investigating co-variates associated with change in the IHS4 score.

Independent Variables	Coefficient	Standard Error	t	*p*-Value
(Constant)	−3.03			
ADDI score	0.16	0.09	1.79	0.0795
Hurley score	0.66	0.11	5.93	<0.0001
Disease duration (years)	0.02	0.01	1.65	0.1055
Gender	0.21	0.18	1.15	0.2579
Change in weight	0.12	0.12	1.00	0.3205

Abbreviations: ADDI (Autoinflammatory Disease Damage Index); HS (hidradenitis suppurativa).

**Table 2:** Multivariate log-linear robust Poisson regression analysis shedding light on the determinants of change in nodules before, during and after the month of Ramadan.

Parameter	B	Standard Deviation	95% CI Wald		Wald’s Chi-Squared	*p*-Value	Exp(B)	95% CI Exp(B)	
			Lower Bound	Upper Bound				Lower Bound	Upper Bound
(Intercecpt)	0.03	0.40	−0.76	0.82	0.00	0.950	1.03	0.47	2.26
No family history of HS	0.62	0.16	0.30	0.93	14.50	0.000	1.85	1.35	2.54
HS phenotype (*vs.* syndromic type)									
Conglobata type	−1.51	0.30	−2.09	−0.92	25.44	0.000	0.22	0.12	0.40
Ectopic type	−1.03	0.28	−1.58	−0.49	13.96	0.000	0.36	0.21	0.61
Frictional foruncle type	−1.33	0.27	−1.87	−0.79	23.57	0.000	0.27	0.16	0.45
Regular type	−0.79	0.23	−1.24	−0.33	11.55	0.001	0.46	0.29	0.72
Scarring folliculitis type	−1.11	0.25	−1.60	−0.62	19.72	0.000	0.33	0.20	0.54
Disease duration	0.02	0.01	−0.01	0.04	2.15	0.143	1.02	1.00	1.04
Hurley score	0.44	0.09	0.25	0.62	21.35	0.000	1.55	1.29	1.86
Change in weight	−0.13	0.11	−0.34	0.08	1.42	0.234	0.88	0.71	1.09

Abbreviations: CI (confidence interval); HS (hidradenitis suppurativa).

**Table 3:** Multivariate log-linear robust Poisson regression analysis shedding light on the determinants of change in abscesses before, during and after the month of Ramadan.

Parameter	B	Standard Deviation	Wald 95% CI		Wald’s Chi-Squared	*p*-Value	Exp(B)	95% CI Exp(B)	
			Lower Bound	Upper Bound				Lower Bound	Upper Bound
(Intercept)	−0.86	0.38	−1.59	−0.12	5.22	0.022	0.42	0.20	0.89
No family history of HS	0.14	0.13	−0.11	0.40	1.18	0.278	1.15	0.89	1.49
HS phenotype (*vs.* syndromic type)									
Conglobata type	0.33	0.22	−0.11	0.76	2.15	0.143	1.38	0.90	2.14
Ectopic type	−0.17	0.51	−1.17	0.83	0.11	0.741	0.84	0.31	2.30
Frictional foruncle type	0.95	0.20	0.56	1.34	22.59	0.000	2.59	1.75	3.83
Regular type	−0.09	0.22	−0.53	0.34	0.18	0.668	0.91	0.59	1.40
Scarring folliculitis type	0.71	0.18	0.36	1.05	15.99	0.000	2.03	1.43	2.87
Non biologics									
Topical antibiotics	−0.47	0.28	−1.02	0.07	2.96	0.085	0.62	0.36	1.07
Systemic antibiotics	−0.08	0.13	−0.34	0.18	0.38	0.539	0.92	0.71	1.20
Hurley score	0.59	0.12	0.36	0.82	25.67	0.000	1.80	1.44	2.27
Change in weight	−0.04	0.06	−0.17	0.08	0.47	0.493	0.96	0.85	1.09

Abbreviations: CI (confidence interval); HS (hidradenitis suppurativa).

**Table 4:** Multivariate log-linear robust Poisson regression analysis shedding light on the determinants of change in draining tunnels before, during and after the month of Ramadan.

Parameter	B	Standard Deviation	Wald’s 95% CI		Wald’s Chi-Squared	*p*-Value	Exp(B)	95% CI Exp(B)	
			Lower Bound	Upper Bound				Lower Bound	Upper Bound
(Intercept)	0.71	0.30	0.11	1.30	5.44	0.020	2.02	1.12	3.66
No family history of HS	−0.01	0.13	−0.26	0.24	0.01	0.928	0.99	0.77	1.27
HS phenotype									
Conglobata type	−0.45	0.24	−0.91	0.02	3.55	0.060	0.64	0.40	1.02
Ectopic type	−0.73	0.20	−1.12	−0.33	12.87	0.000	0.48	0.33	0.72
Frictional foruncle type	−1.19	0.16	−1.51	−0.87	53.30	0.000	0.30	0.22	0.42
Regular type	−0.77	0.17	−1.11	−0.43	20.17	0.000	0.46	0.33	0.65
Scarring folliculitis type	−1.29	0.16	−1.60	−0.97	64.93	0.000	0.28	0.20	0.38
Non biologics									
Topical antibiotics	−0.05	0.19	−0.43	0.33	0.06	0.804	0.95	0.65	1.39
Systemic antibiotics	0.07	0.12	−0.16	0.30	0.37	0.543	1.07	0.85	1.35
Hurley score	0.23	0.09	0.05	0.41	6.35	0.012	1.26	1.05	1.51
Change in weight	0.09	0.09	−0.08	0.25	0.98	0.322	1.09	0.92	1.29

Abbreviations: CI (confidence interval); HS (hidradenitis suppurativa).
